# Epidemic intelligence data of Crimean-Congo haemorrhagic fever, European Region, 2012 to 2022: a new opportunity for risk mapping of neglected diseases

**DOI:** 10.2807/1560-7917.ES.2023.28.16.2200542

**Published:** 2023-04-20

**Authors:** Angela Fanelli, Johannes Christof Schnitzler, Marco De Nardi, Alastair Donachie, Ilaria Capua, Gianvito Lanave, Domenico Buonavoglia, Paula Caceres-Soto, Paolo Tizzani

**Affiliations:** 1 Department of Veterinary Medicine, University of Bari, Bari, Italy; 2 One Health Center of Excellence, University of Florida, Gainesville, Florida, United States; 3 Intelligence Innovation and Integration unit, World Health Organization, Berlin, Germany; 4 SAFOSO AG, Liebefeld, Switzerland; 5 World Animal Health Information and Analysis Department, World Organisation for Animal Health, Paris, France

**Keywords:** epidemic intelligence, EIOS, digital data, Crimean-Congo haemorrhagic fever, risk mapping

## Abstract

**Background:**

The Epidemic Intelligence from Open Sources (EIOS) system, jointly developed by the World Health Organisation (WHO), the Joint Research Centre (JRC) of the European Commission and various partners, is a web-based platform that facilitate the monitoring of information on public health threats in near real-time from thousands of online sources.

**Aims:**

To assess the capacity of the EIOS system to strengthen data collection for neglected diseases of public health importance, and to evaluate the use of EIOS data for improving the understanding of the geographic extents of diseases and their level of risk.

**Methods:**

A Bayesian additive regression trees (BART) model was implemented to map the risk of Crimean-Congo haemorrhagic fever (CCHF) occurrence in 52 countries and territories within the European Region between January 2012 and March 2022 using data on CCHF occurrence retrieved from the EIOS system.

**Results:**

The model found a positive association between all temperature-related variables and the probability of CCHF occurrence, with an increased risk in warmer and drier areas. The highest risk of CCHF was found in the Mediterranean basin and in areas bordering the Black Sea. There was a general decreasing risk trend from south to north across the entire European Region.

**Conclusion:**

The study highlights that the information gathered by public health intelligence can be used to build a disease risk map. Internet-based sources could aid in the assessment of new or changing risks and planning effective actions in target areas.

**Key public health message**

**What did you want to address in this study?**

Epidemic Intelligence from Open Sources (EIOS) initiative provides a means of disseminating information among public health agencies. In this study, we investigated whether online information retrieved from the EIOS can be used to produce a risk map for Crimean-Congo haemorrhagic fever (CCHF) within the European Region. CCHF is a tick-borne disease caused by a virus belonging to the *Nairoviridae* family.

**What have we learnt from this study?**

Using CCHF as a test study, we learnt that epidemic intelligence tools like EIOS can be used to automatically extract information on outbreak locations, which may, in turn, be used to identify the areas at high risk of disease occurrence.

**What are the implications of your findings for public health?**

Online data on the risk of disease in different locations can be used by decision makers, public health authorities as well as veterinary services to identify areas where surveillance is most needed.

## Introduction

The Epidemic Intelligence from Open Sources (EIOS) [1] platform is a unique collaboration between various public health stakeholders around the globe. The initiative is led by the World Health Organization (WHO) and brings together new and existing networks and systems to create a unified all-hazards, One Health approach to early detection, verification and assessment of public health risks and threats. The EIOS system, jointly developed under the EIOS initiative by WHO, the Joint Research Centre (JRC) of the European Commission and partners is a web-based platform that enables monitoring of information in near real-time from around 15,000 publicly accessible sources worldwide (e.g. news media, social media, scientific papers), using specific search algorithms. EIOS promotes and catalyses new and innovative collaborative development, appealing and connecting several actors, institutions and countries [2]. 

Information collated from open sources is automatically processed, analysed and enriched with metadata including classifications based on more than 600 categories representing different concepts (for example: diseases, variants, public health measures). Other meta information relevant for this study is the detection of mentioned geolocations. Geolocations are identified using named entity recognition with disambiguation (NEROne) [3], mapped to the geographical database GeoNames [4].

In order to facilitate detection of new relevant threats or monitoring of ongoing threats, the EIOS system provides the user with several filter options to focus on, for the given user, most relevant information. Specifically, data can be filtered by time period, mentioned countries, geolocations, region, language, source of the data, as well as categories. All these filters can be combined to create a tailored search and filter capabilities which can be saved. These characteristics positioned the EIOS as one of the most relevant systems for event-based surveillance [5].

EIOS collects information from a wide range of unofficial and official sources (e.g. official channels from national authorities) on animal and human diseases. The World Organisation for Animal Health (WOHA) and the WHO, organisations that disseminate officially confirmed information on animal and human diseases respectively, have different mechanisms and systems for collecting and disseminating this official information. In the field of animal health, the World Animal Health Information System (WAHIS) is the WOAH reference global database that gathers information on the official animal disease situation as reported by the National Veterinary Services [6]. This database has been proved to be particularly useful to understand the evolution of animal diseases at global and regional level [7-12]. Information on human health is available through different mechanisms, with varying coverage, completeness, and update frequency (accessible for example through the Global Health Observatory [13], the Global Influenza Programme [14], Disease Outbreak News [15] among others). Data from media sources, expert networks and social media can be pivotal to identify significant disease outbreak information in a near real-time way [16], but they also represent critical sources to understand the spatio-temporal distribution of diseases when official data are lacking. Nevertheless, information from unofficial sources needs to be verified and confirmed.

In this study, we used internet-based data retrieved from the EIOS system to model the risk of Crimean-Congo haemorrhagic fever (CCHF) occurrence in the European Region. The objectives were (i) to assess the capacity of the EIOS system to strengthen data collection for neglected diseases of veterinary and public health importance and (ii) to evaluate the use of the EIOS data for improving the understanding of the geographic extents of diseases and their level of risk. CCHF is a tick-borne disease caused by the arbovirus Crimean-Congo haemorrhagic fever virus (CCHFV; family *Nairoviridae*) transmitted by ticks of the genus *Hyalomma* [17-20]. A previous study attempted to model the risk of CCHF infection in humans using reported cases in literature [21]. However, thus far, no study has used epidemic intelligence tools investigating publicly available sources to create a database of occurrence points with the view of building a disease risk map.

## Methods

### Geographical setting and definitions

The study area is represented by the territories of 52 countries and territories within the European Region below ~60 ^◦^North latitude (see Supplementary Material A, Table S1 for a list of the countries and territories considered in this study). 

An occurrence point was defined as a geographical point where a case of CCHF in humans or an isolation of CCHFV from ticks was detected during the timeframe considered. This latitude was defined considering the gaps in satellite normalised difference vegetation index (NDVI) coverage at regions near the poles because of the long-lasting accumulation of snow or clouds, as well as the stable presence of the CCHF vector (ticks of genus *Hyalomma*) [22,23].

### Data sources and approach

Data on CCHF occurrence (January 2012–March 2022) were retrieved from the EIOS system using a specific category (‘CCHF category’) that implemented an algorithm to detect mentions of CCHF cases in humans or vectors. In practice, a list of predefined keywords and keyword combinations in different languages was used (see Supplementary Material B for an example of keywords in English). The EIOS system collates information from a broad range of sources including news media, social media and expert networks and news aggregator websites (e.g. ProMed, HealthMap and the Global Public Health Intelligence Network (GPHIN). The EIOS system is constantly updated, collecting ‘close to real-time’ events. Data included in this study have incorporated the information available up to 2 March 2022. Since EIOS only started a consistent data retrieval in November 2017, we coupled these data with the information on CCHF cases during the period 2012–17 obtained from scientific literature.

The approach was based on the following steps: (i) building a filter in EIOS to detect all the news items falling under ‘CCHF category’, (ii) retrieving and double checking the geographical coordinates detected in the news items by EIOS, (iii) assessing the reliability of the data, (iv) complementing the occurrence data extracted from EIOS with the information retrieved from scientific literature, (v) retrieving and processing the rasters used as predictors and (vi) building a spatial model. Details of the methodology applied are provided hereunder.

### Filter criteria and data processing 

Firstly, a filter was created in EIOS to identify and pin all news items that mentioned CCHF cases in humans or vectors to the ‘CCHF category’. Secondly, a spatial selection was applied, and a target area was drawn on the interface map provided by the system to include only the information relevant to the European Region. Lastly, each piece of news, along with its ID (assigned by EIOS) and geographical coordinates of all the mentioned locations, was extracted and exported to Excel. If a news item contained information about more than one location of the disease, multiple rows were created in the export. Each news item was thoroughly checked manually by the authors (AF, JCS and PT) to assess (i) the credibility and reliability of the website, (ii) the content of the news item, (iii) the accuracy and reliability of the source, (iv) confirmation of the same news item by more than one source and (v) strength of evidence for CCHF presence in that location. To do so, the map on geographic distribution of CCHF developed by WHO showing the areas with active CCHF circulation (virological and serological evidence) was consulted as one of the criteria to check and validate the information retrieved [24]. News items mentioning the locations where the cases were hospitalised rather than the locations of the infection were discarded. Similarly, we did not consider news items where it was not possible to distinguish the infection site from the hospital location. 

Given the limitations of the EIOS system before 2017, the dataset was completed by including information on CCHF cases retrieved from studies published after 2012 (mentioned in a recent meta-analysis [25]). To better define the territorial entities, two columns were added to the database, one containing the GeoNames Feature Code retrieved from GeoNames website [4] and another containing the information on the area size (expressed as km^2^) of the territorial entity and retrieved from Wikipedia. After removal of duplicates (news items and information from scientific literature referring to more cases occurring in the same location), administrative divisions greater than 5,000 km^2^ were not included in the analysis to reduce the risk of bias in the model. If we had included large areas of presence, the predictors would have needed to be scaled at a lower resolution. The use of highly resolved predictors is indeed an important prerequisite of spatial modelling since the detail of disease response to the environments may not be captured at a coarser resolution.

### Predictor variables

In this study, the term pixel is used to define the level of detail (cell size or spatial resolution) of the rasters used as predictors in the model. Thirty-three explanatory variables were initially considered to build the model (Table). We used the functions in the 'sdmpredictors' package [26] to access WorldClim [27] and ENVIREM [28] online datasets. For the NDVI, we downloaded the 10-daily data PROBAV_V2.2.1 (1 Jan 2016–31 Dec 2020) from Copernicus Global Land Service [29]. One-month composites of NDVI were prepared through the method of the maximum pixel value to obtain the largest area without gaps in pixels. Principal component analysis (PCA) was run on the monthly composites using the prcomp() function to reduce the number of NDVI rasters. The first four components explaining 0.91% of the total variance were added to the set of explanatory variables (see Supplementary Material A, Figure S1 A for an analysis of the first four principal components). All the predictors were rescaled at a resolution of 0.044645 x 0.044645 decimal degrees (corresponding to around 4 x 4 km in metric units), aligned and reprojected using the same coordinate reference system (World Geodetic System 1984 (WGS84) EPSG:4326).

**Table:** Variables collated for use in the spatial model on Crimean-Congo haemorrhagic fever occurrence in 52 countries and territories within the European Region, 2012–2022

Definition	Code	Source
Annual mean temperature	WC_bio1	WorldClim [27]
Mean diurnal range (mean of monthly (max temperature − min temperature))	WC_bio2	WorldClim [27]
Isothermality (bio2/bio7) (x 100)	WC_bio3	WorldClim [27]
Temperature seasonality (standard deviation x 100)	WC_bio4	WorldClim [27]
Max temperature of the warmest month	WC_bio5	WorldClim [27]
Min temperature of the coldest month	WC_bio6	WorldClim [27]
Temperature annual range (bio5 –bio6)	WC_bio7	WorldClim [27]
Mean temperature of the wettest quarter	WC_bio8	WorldClim [27]
Mean temperature of the driest quarter	WC_bio9	WorldClim [27]
Mean temperature of the warmest quarter	WC_bio10	WorldClim [27]
Mean temperature of the coldest quarter	WC_bio11	WorldClim [27]
Annual precipitation	WC_bio12	WorldClim [27]
Precipitation of the wettest month	WC_bio13	WorldClim [27]
Precipitation of the driest month	WC_bio14	WorldClim [27]
Precipitation seasonality (coefficient of variation)	WC_bio15	WorldClim [27]
Precipitation of the wettest quarter	WC_bio16	WorldClim [27]
Precipitation of the driest quarter	WC_bio17	WorldClim [27]
Precipitation of the warmest quarter	WC_bio18	WorldClim [27]
Precipitation of the coldest quarter	WC_bio19	WorldClim [27]
Thornthwaite aridity index: index of the degree of water deficit below water need	ER_aridityIndexThornthwaite	ENVIREM dataset [28]
A metric of relative wetness and aridity	ER_climaticMoistureIndex	ENVIREM dataset [28]
Average temperature of the warmest month − average temperature of the coldest month	ER_continentality	ENVIREM dataset [28]
Sum of mean monthly temperature for months with mean temperature greater than 0 °C multiplied by number of days	ER_growingDegDays0	ENVIREM dataset [28]
Sum of mean monthly temperature for months with mean temperature greater than 5 °C multiplied by number of days	ER_growingDegDays5	ENVIREM dataset [28]
Count of the number of months with mean temperature greater than 10 °C	ER_monthCountByTemp10	ENVIREM dataset [28]
Compensated thermicity index: sum of mean annual temperature, min temperature of the coldest month, max temperature of the coldest month x 10, with compensations for better comparability across the globe	ER_thermicityIndex	ENVIREM dataset [28]
SAGA GIS topographic wetness index	ER_topoWet	ENVIREM dataset [28]
Terrain roughness index	ER_tri	ENVIREM dataset [28]
Principal component 1 of NDVI^a^ time series	ndvi.pca1	Copernicus Global Land Service [29]
Principal component 2 of NDVI^a^ time series	ndvi.pca2	Copernicus Global Land Service [29]
Principal component 3 of NDVI^a^ time series	ndvi.pca3	Copernicus Global Land Service [29]
Principal component 4 of NDVI^a^ time series	ndvi.pca4	Copernicus Global Land Service [29]

max: maximum; min: minimum; NDVI: normalised difference vegetation index.

^a^ NDVI is an indicator of the greenness of the biomes.

A list of the countries and territories included can be found in Supplementary Material A Table S1 A.

### Occurrence points and pseudo-absence data

A spatial thinning procedure selecting only one presence within each pixel of the predictor variables was performed with gridRecords() function in fuzzySim package version 3.6 [30] to reduce both spatial bias and spatial autocorrelation. This function was used to obtain unique presences and absences from occurrence data (expressed as x and y coordinates) at the spatial resolution of the predictors. As absence points were not supplied, all pixels without any occurrence points were returned as pseudo-absences. Nevertheless, a random sample of the pseudo-absence pixels was selected to obtain a 1:10 ‘occurrence: pseudo-absence ratio’ in the model. This was done to avoid an excessive number of pseudo-absences compared with the number of occurrences [30].

### Spatial modelling

We built a Bayesian additive regression tree (BART) model using the embarcadero package version 1.2.0.1003 [31]. Under the Bayesian framework, the initial expected value (prior) is updated by the model to get the posterior distribution (predicted probabilities). Thus, for each pixel, a posterior distribution of predicted probabilities with the associated 95% credible intervals (CI) is obtained. The model was fit with all the variables to select the most important ones. The automated stepwise reduction algorithm with 50 iterations and 10 trees was used to eliminate the variables with the lowest importance and obtain the model with the lowest root mean square error (RMSE). Afterwards, the model was rerun using only the variables selected in the previous step. Variable importance plot, predictions and response plots were created with the in-built functions of the package. Finally, the probability maps were converted to favourability maps using modEVA package version 3.0 [32]. The percentage of pixels at different levels of risk and uncertainty were computed reclassifying the pixels values into three classes, i.e. low (0 ≤ x < 0.33), medium (0.33 ≤ x < 0.66), and high (0.66 ≤ x), with QGIS version 3.22.4 [33].

Model performance was assessed by the area under the receiver operating characteristic curve (AUC), which measures overall discrimination capacity and threshold-dependent metrics and assesses how well the model distinguishes presence from absence. All the analyses were performed R software version 4.1.2 [34]. 

## Results

**Figure 1:**
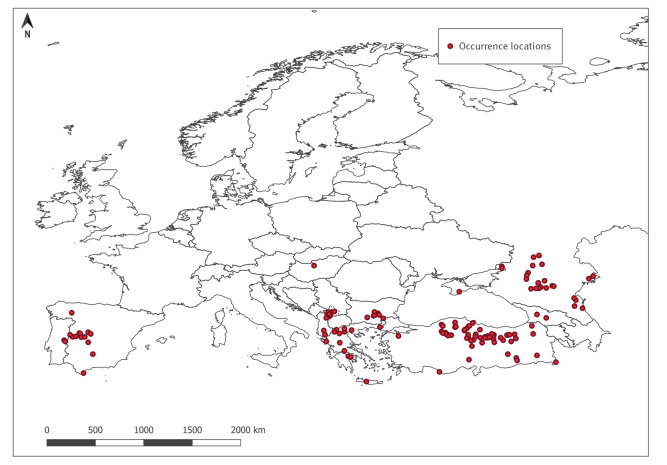
Occurrence locations of Crimean-Congo haemorrhagic fever cases in humans and Crimean-Congo haemorrhagic fever virus isolates from ticks, European Region, 2012–2022 (n = 141 before spatial thinning)

Three hundred and sixty-five news items published between 24 July 2012 to 22 March 2022 were retrieved from the EIOS system. These comprised 1,387 locations. Fifty-one additional locations were compiled from the meta-analysis. After removing the EIOS duplicates, the irrelevant data (news items not specifically referring to human cases or virus isolation from ticks) and uncertain information (news items without clear indication of the localisation of human cases/virus isolation from ticks), we obtained 172 records, of which further 31 were discarded as referring to administrative divisions with areas greater than 5,000 km^2^. Thus, 141 occurrence points were initially considered (Figure 1).

**Figure 2:**
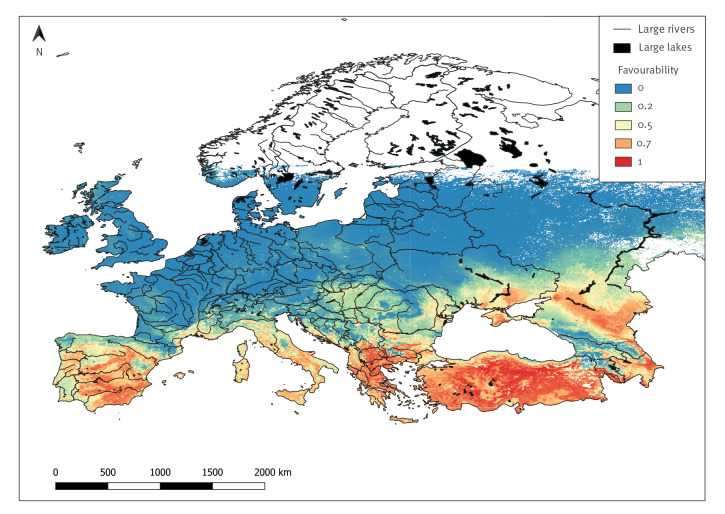
Favourability predictions for Crimean-Congo haemorrhagic fever occurrence, European Region below ~60 ^◦^North latitude, 2012–2022

Following the spatial thinning, the final occurrence dataset comprised 136 pixels with occurrence and 1,360 pixels with pseudo-absence of CCHF. The model with the lowest average RMSE selected through the automated procedure retained the following variables in order of importance: ER_growingDegDays0, ndvi.pca1, ER_aridityIndexThornthwaite, WC_bio9, ER_continentality, WC_bio5, ER_tri, and WC_bio11 (see Supplementary Material A, Figure S2 A and S3 A for the variable selection and variable importance plots respectively). Only the first principal component of the NDVI was selected. This captures the major element of variability in the NDVI time series. The model performed well in terms of accuracy (AUC = 0.95) (see Supplementary Material A, Figure S4 A for the model diagnostics), correct classification rate (CCR = 0.80), correct prediction of presences (sensitivity/recall = 0.99), correct prediction of absences (specificity = 0.79), true skill statistic (sTSS = 0.89), Cohen’s kappa (skappa = 0.69), but the positive predictive value was low (precision = 0.31) (see Supplementary Material A, Figure S5 A for the model evaluation metrics). Figure 2 shows that the majority of CCHF risk is found in the Mediterranean basin and in the areas bordering the Black Sea. Considering the whole study area, the percentage of pixels at high risk was 16%, while 15% and 69% were at medium and low risk, respectively. There was a general decreasing risk trend from south to north across the entire European Region.

**Figure 3:**
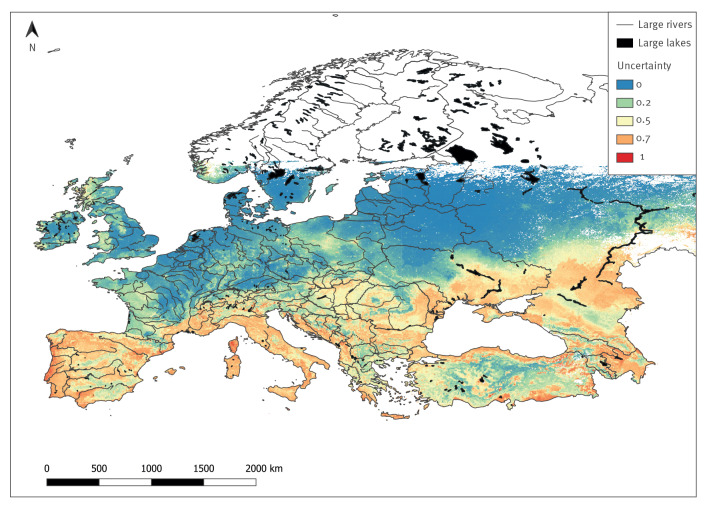
Uncertainty predictions^a^ for Crimean-Congo haemorrhagic fever occurrence, European Region below ~60 ^◦^North latitude, 2012–2022

^a^ Posterior width, 95% credible intervals.

The uncertainty map highlights that some areas of predicted favourability are characterised by considerable uncertainty, presumably because of the lack of CCHF records in those zones (Figure 3). Nevertheless, the majority of the study area is characterised by a low level of uncertainty (57% of pixels), while only a smaller percentage of pixels are characterised by either medium (37%) or high (6%) levels of uncertainty.

**Figure 4:**
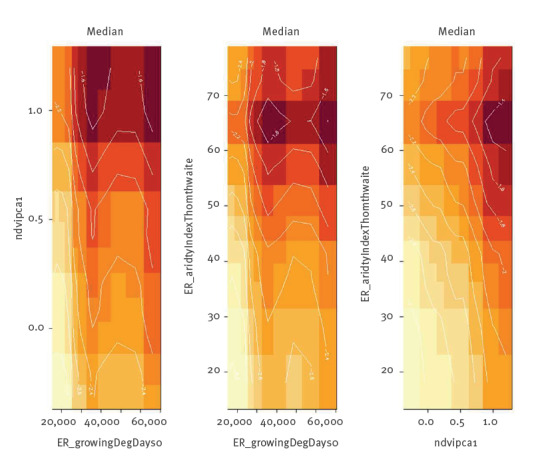
Bidimensional partial dependence plots of three variables^a^ showing the interactions between pairs of variables influencing Crimean-Congo haemorrhagic fever occurrence probability, European Region below ~60 ^◦^North latitude, 2012–2022

^a^ Bidimensional plots for ndvi.pca1 (principal component 1 of NDVI time series), ER_growingDegDays0 (sum of mean monthly temperature for months with mean temperature greater than 0 °C multiplied by number of days) and ER_aridityIndexThornthwaite (Thornthwaite aridity index: index of the degree of water deficit below water need). NDVI is an indicator of the greenness of the biomes.

Considering the variables retained, the model found a positive association between all temperature-related variables and the probability of CCHF occurrence, with an increased risk in warmer and drier areas (see Supplementary Material A, Figure S6 A for the partial dependence plots). In the model, there was no predictor that alone showed a marked and significant effect, but rather the combination of all variables influenced the risk of CCHF across the European Region. Considering the bidimensional partial dependence plots for the first three selected variables (Figure 4), the occurrence probability is remarkably high in those areas characterised by both high values of ER_growingDegDays0 and ndvi.pca1, both high values of ER_growingDegDays0 and ER_aridityIndexThornthwaite, and both high values of ndvi.pca1 and ER_aridityIndexThornthwaite.

The occurrence probability is represented by the colour progression: from white (lowest probability) to dark red (highest probability).

## Discussion

In this study, we used internet-based surveillance data retrieved from the EIOS system to model the risk of CCHF occurrence within the European Region. The study underlines the effectiveness of the EIOS system in detecting, retrieving and compiling information from multiple sources and how these data can be used to build a disease risk map. CCHF was considered a good test case for several reasons. Firstly, the disease, which is endemic in many regions such as Africa, Asia, Eastern Europe and the Middle East, has given rise to many concerns over its spread outside the current geographical range [18]. Secondly, it is a tick-borne disease, and thus environmental variables play a pivotal role in shaping its geographical distribution and consequently its occurrence, can be well predicted through spatial modelling [19]. Thirdly, it is a neglected tropical disease posing a risk to human health [20]. Results of the model highlight that the distribution of CCHF is likely to be geographically broader than expected, and mainly driven by temperature/climate-related predictors. Not only does the model predict areas where CCHF is already established (e.g. Eastern Europe and the Black Sea region), but also those areas at risk of further CCHF expansion, given their favourable environmental conditions (e.g. Italy, France). Of note, during the revision of this work, author Fanelli and colleagues found seropositive cattle in Italy [35], confirming model predictions. It is important to stress that the presence probability predictions were converted into favourability predictions. The former are considered as the likelihood of finding the disease in a given environment depending on the probability of finding the disease given how rare or common it is (i.e. prevalence), whereas the latter do not necessarily reflect the locations where the disease is predicted to be present, but rather favourable areas for its occurrence, although the disease may be currently absent [36]. It is not surprising that the model has a low precision, meaning that it predicts positive pixels where we do not have any actual observations of human cases or virus isolation from ticks. These are represented by the locations which are likely to be favourable for CCHF occurrence, but where the disease or the circulation of the virus have yet to be recorded.

Another important consideration highlighted by this study is related to the countries where the disease seems to have a clustered distribution for which a low favourability and high uncertainty were found. One example is Georgia and its vicinity, where the presence points retrieved from the news items were limited to the plain areas of Khashuri and Tskaltubo. The low favourability and high uncertainty found in the areas close to the plain is not surprising given the complex orographic and very variable landscape-climatic conditions of the area. The low favourability is likely to be due to the mountain areas (Caucasus mountains) with lower temperatures and mostly unfavourable environmental conditions.

The overall percentage of areas at high favourability is quite low, highlighting that the risk is still low for most European countries. Interestingly, our predictions are in line with the risk estimates of authors Fanelli and Buonavoglia in an earlier publication [17]. In their assessment, a low risk of CCHFV entry and exposure in large part of Western Europe and a medium risk for France and Italy was described. The presence of the *Hyalomma marginatum* resident population, which is the main vector of the virus, is the primary factor influencing the probability of the disease spread once the virus has been introduced in CCHF-free countries [17]. In our predictive map, the risk is high for almost the entire Italian peninsula, whereas France has the highest favourability along its Mediterranean coast. In line with this, Italy has a widespread population of *H. marginatum,* whereas France has only a hotspot area in the southern part of the country [22].

Fernández-Ruiz and Estrada-Peña [19] have recently assessed the expansion of suitable areas for *H. marginatum*, indicating that the Mediterranean countries, south-east central Europe and the southern Balkans are most likely to see future spread of the tick. Similarly, in our study, the most important variables retained by the model define the most favourable areas for the spread and colonisation of *H. marginatum.* It is important to derive the ecological meaning of the predictors retained, which are likely to play an important role in determining the suitability of areas for tick development. A relevant example is the sum of mean monthly temperature for months with mean temperature greater than 0 °C multiplied by number of days, which may define the critical accumulated temperature necessary for tick spread and colonisation [19]. Another important predictor in our model is the NDVI which, at high values, contributes, along with the temperature variables, to increasing the probability of CCHF occurrence, which may be explained by the necessity of adequate relative humidity for the vector development [23].

In this study, we did not include any data on CCHF serology in humans or animals, which are unlikely to be reported in near real-time news item publications, as are the data of the EIOS system. The information on serological evidence of CCHF comes mainly from scientific literature and may be useful to complement the picture of the disease distribution [16]. It is worth mentioning that CCHFV can circulate unnoticed in numerous wild and domestic animals that are asymptomatic carriers of the virus and may act as reservoirs [37]. Cuadrado-Matías et al. [38] mapped the risk of exposure to CCHFV in Spain using serological data on red deer as an indicator of the transmission risk from infected ticks. Compared with their risk map, our predictions show a more extensive distribution of favourable environment in the Iberian Peninsula for CCHF occurrence. In particular, they found a higher risk mainly in the south-western part of the country, whereas our predictions indicate that the risk is high also along the Spain's east coast. This difference is likely to be due to the different modelling approaches adopted as well as the predictors used. Indeed, not only did Cuadrado-Matías et al. [38] use environmental variables, but also host population predictors regarding both wild and domestic animals, which were found to be significant factors behind the risk of exposure to CCHFV. Unfortunately, this information, which could have improved our predictions, is missing or outdated at the European level. With reference to the distribution and density of domestic animals, some data are available through the Gridded Livestock of the World (GLW3) from the Food and Agriculture Organization of the United Nations (FAO), but the dataset has two major limitations: the distribution data refer to 2010 and their spatial resolution is around 10 km (coarser than our predictions) [39]. Wildlife data are available for most of the European countries, but they are not collected with consistent and homogeneous criteria, making it very difficult to use for modelling purposes [40].

It is also important to highlight that the model was run on the full dataset (resubstitution). Indeed, it was not possible to set aside some data for testing given the small amount of presence points. Data splitting would have withdrawn a lot of information from the model. This should be kept in mind when considering the results of this study. Other limitations include the category definition for CCHF available in EIOS, which might have missed some news items reporting CCHFV from ticks. Despite these limitations, we feel that our model is of great value since it provides an updated picture of the risk of CCHF at the regional level, with a low level of uncertainty associated with the majority of the pixels. 

Our approach is unique in its kind, as we demonstrated the potential of internet-based data accessed through the EIOS system to create a disease risk map. In fact, although the traditional use of the EIOS system is to early detect potential public health threats through an event-based surveillance [41-43], their complex algorithms can be also used to automatically extract the geographical coordinates of outbreak locations, which may, in turn, be used to build spatial risk models. These have important consequences for health-related decision-making and planning effective actions in target areas [44-49].

## Conclusions

The practical implications of using epidemic intelligence tools are considerable in terms of helping design effective disease control strategies. However, they require a reasonable effort to develop adequate and unbiased algorithms, to select accurate and relevant sources, and to extract the exact geographical coordinates of disease outbreaks. 

As recently mentioned in a paper on the ‘costs and benefits of primary prevention of zoonotic pandemics’ [50], current plans to address future pandemic catastrophes still mainly consider the approach of ‘detecting and containing emerging zoonotic threats’ [51]. However, much more must be done on the prevention side. Reacting to a spillover/pandemic/epidemic event after it occurs can be, in fact, much more costly than working to prevent it. In this sense, the case study presented here may constitute a model to combine epidemic intelligence tools and advanced analytical approaches to detect and assess changes in disease distribution, allowing prevention or early mitigation of disease events of veterinary and public health importance.

## Supplementary Data

293000 Supplement
