# Supplementary material for: Epidemic intelligence data of Crimean-Congo haemorrhagic fever, European Region, 2012 to 2022: a new opportunity for risk mapping of neglected diseases
Source: Euro Surveill. 2023 Apr 20;28(16):2200542. doi: 10.2807/1560-7917.ES.2023.28.16.2200542 (PMC10283452; doi:10.2807/1560-7917.ES.2023.28.16.2200542)
Supplement: Supplement [file 22-00542_FANELLI_SUPPLEMENT.pdf]

This supplementary material is hosted by Eurosurveillance as supporting information alongside the article "Epidemic intelligence data of CCHF, European Region 2012-2022: a new opportunity for risk mapping of neglected diseases" on behalf of the authors who remain responsible for the accuracy and appropriateness of the content. The same standards for ethics, copyright, attributions and permissions as for the article apply. Supplements are not edited by Eurosurveillance and the journal is not responsible for the maintenance of any links or email addresses provided therein

Supplementary material A: Details of the study

Table S1 A: European countries and territories below ~ 60°North latitude considered in this study

**Country name**
Albania
Andorra
Armenia
Austria
Azerbaijan
Belarus
Belgium
Bosnia and Herzegovina
Bulgaria
Croatia
Czech Republic
Denmark
Estonia
Faeroe Islands (Denmark)
Finland
France
Georgia
Germany
Gibraltar (UK)
Greece
Guernsey (UK)
Hungary
Iceland
Ireland
Isle of Man (UK)
Italy
Jersey (UK)
Latvia
Liechtenstein
Lithuania
Luxembourg
Malta
Monaco
Montenegro
Netherlands
North Macedonia

Norway
Poland
Portugal
Republic of Moldova
Romania
Russian Federation
San Marino
Serbia
Slovakia
Slovenia
Spain
Sweden
Switzerland
Türkiye
Ukraine
United Kingdom of Great Britain and Northern Ireland

Figure S1 A: Principal component analysis of the Normalized Difference Vegetation Index (NDVI). PC 1 to 4 represents the first 4 components from PCA, explaining 0.91% of the total variance of NDVI

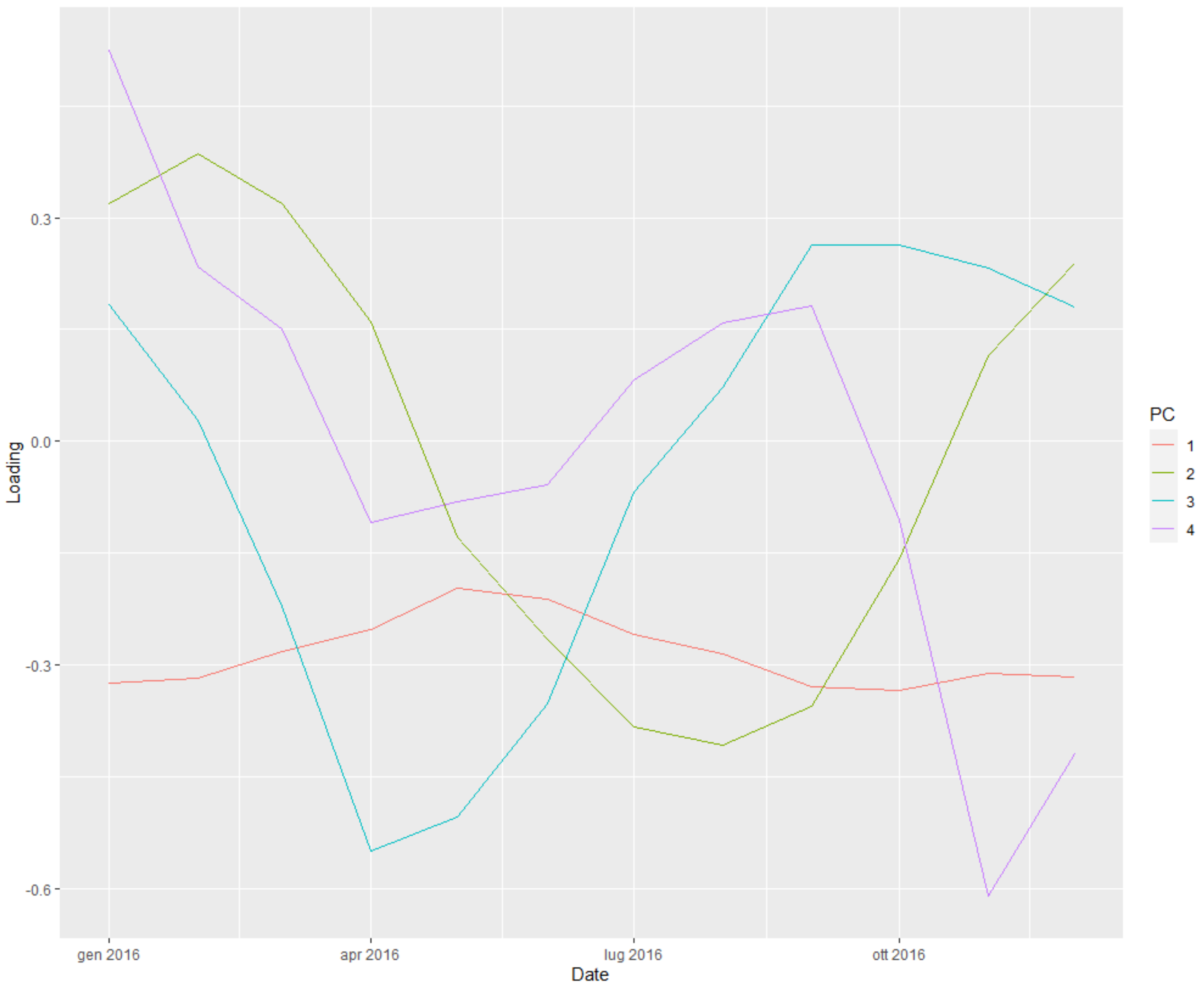

Figure S2 A: Diagnostic of variable importance at different *m* values

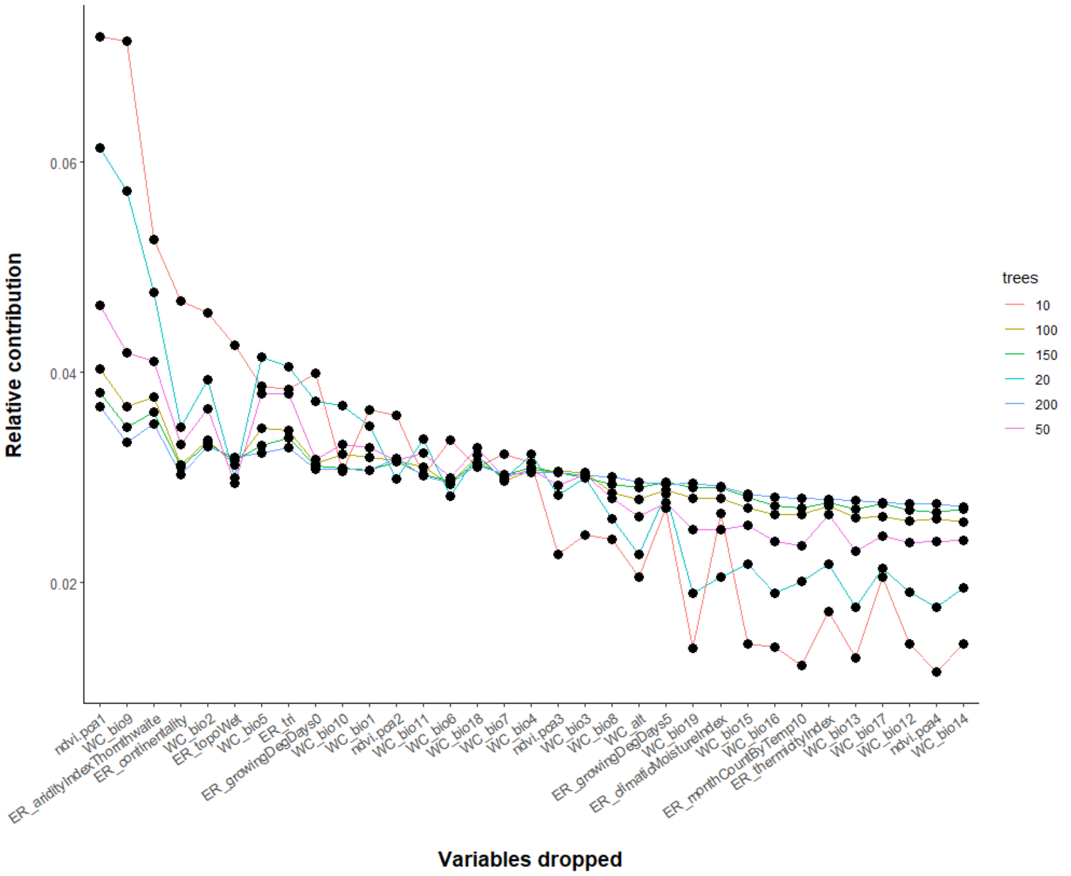

Figure S3 A: Variable importance in the CCHF risk model

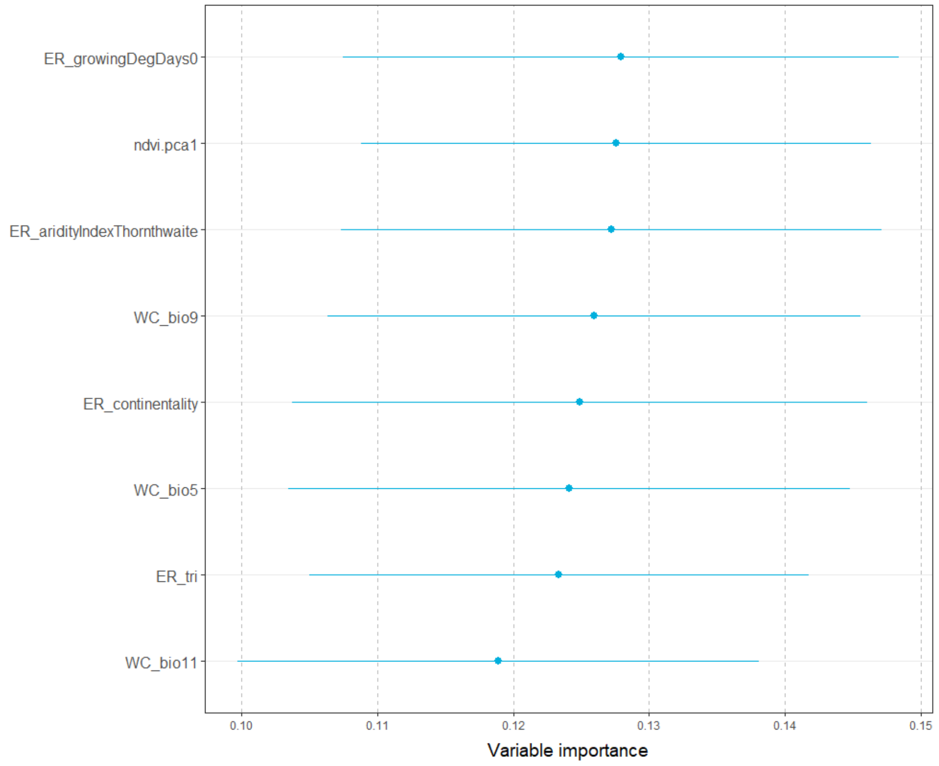

Figure S4 A: Summary model diagnostics

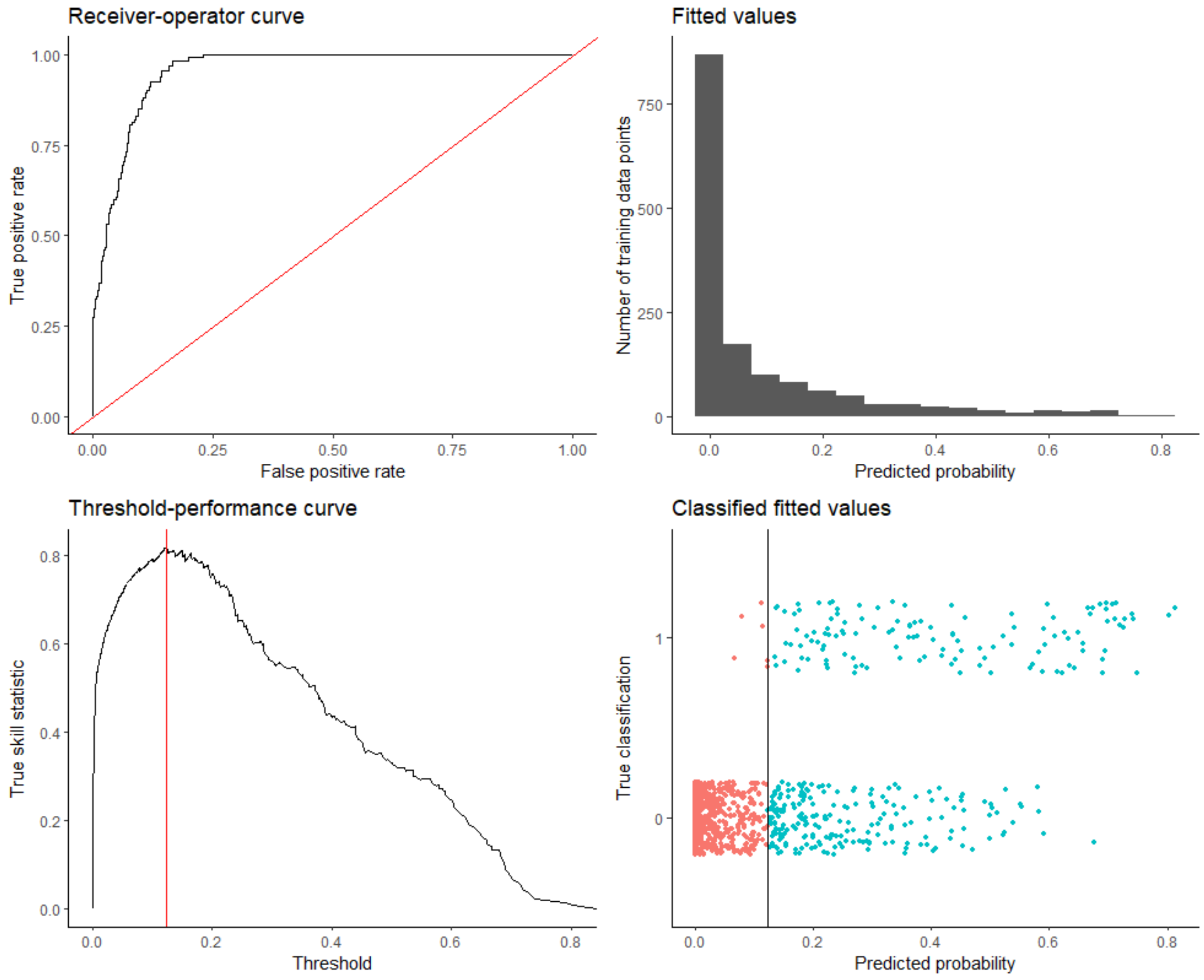

Figure S5 A: Model evaluation metrics

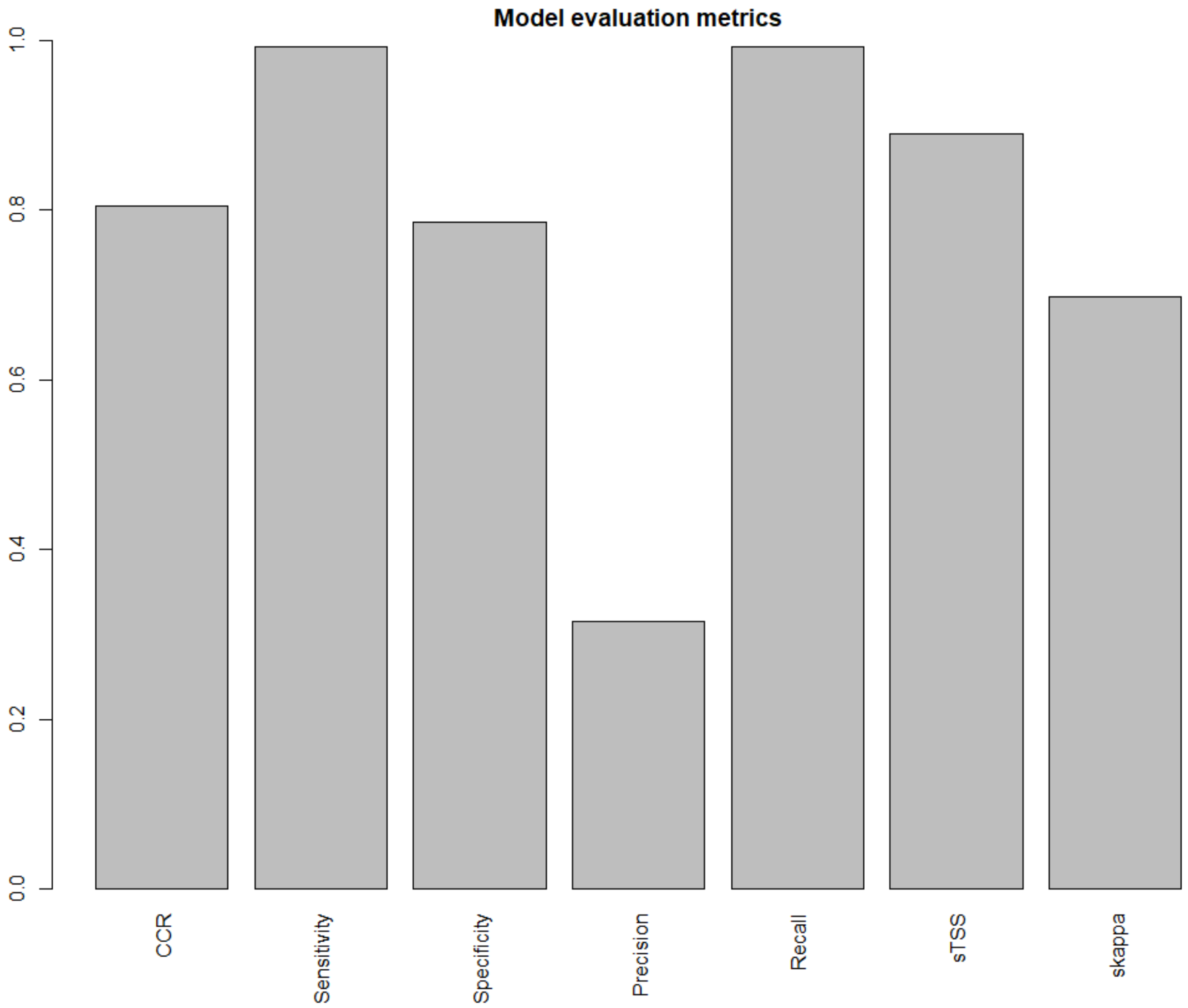

Figure S6 A: Partial dependence plots

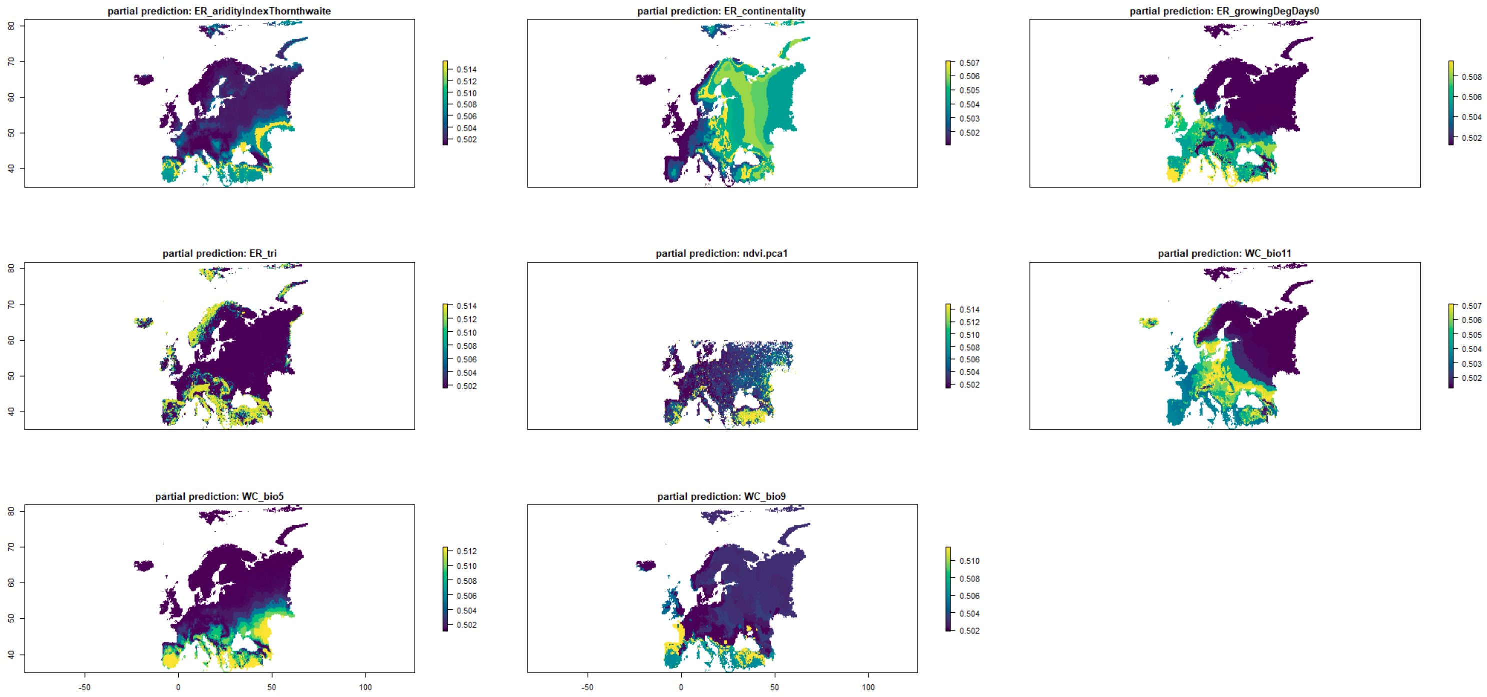

Supplementary material B: Filter criteria illustration

For illustration: Example of lexical rules in English

**Pattern list**
**Positive list**
congo+fever
crimean+congo
crimean-congo
congo-crimean+haemorrhagic+fever
crimean+congo+haemorrhagic+fever
haemorrhagic+crimean+fever
crimean+congo+hemorrhagic+fever
cchf
**Negative list**
film%
movie%
citizens+council+for+health+freedom
c%+c%+h%+foundation%
chronic+congestive+heart+failure
cheap+car+hire+finder
christian+community+health+fellowship

Pattern list

Positive list: List of terms that if found will result in the article being tagged with CCHF.

Negative list: List of terms that if found will result in the article not being tagged with CCHF.

Result of the negative list overrides the result of the positive list.

Wild card characters used in the example

Percent (%)

Used in the place of 0, 1 or more characters.

Plus (+)

Represents white space, such as a space between two words.
